# Comparative Mitogenomics of Two Sympatric Catfishes of *Exostoma* (Siluriformes: Sisoridae) from the Lower Yarlung Tsangpo River and Its Application for Phylogenetic Consideration

**DOI:** 10.3390/genes13091615

**Published:** 2022-09-08

**Authors:** Zheng Gong, Wanxiang Jiang, Huizhe Feng, Yanchao Liu, Tianshun Zhu

**Affiliations:** 1 College of Life Sciences, Zaozhuang University, Zaozhuang 277160, China; 2 Key Laboratory of Ecological Impacts of Hydraulic-Projects and Restoration of Aquatic Ecosystem of Ministry of Water Resources, Institute of Hydroecology, Ministry of Water Resources and Chinese Academy of Sciences, Wuhan 430079, China; 3 Tibet Plateau Institute of Biology, Lhasa 850000, China

**Keywords:** mitochondrial genome, *Exostoma*, Glyptosternae, phylogeny, divergence time, selective pressure

## Abstract

The genus *Exostoma* is a group of stenotopic and rheophilic glyptosternine catfishes distributed in South and Southeast Asia. So far, comprehensive studies on mitogenomics referring to this genus are very scarce. In this study, we first sequenced and annotated the complete mitochondrial genomes of *Exostoma tibetanum* and *Exostoma tenuicaudatum*—two sympatric congeners from the lower Yarlung Tsangpo River, Tibet, China. The mitogenomes of both species contained 13 protein-coding genes, two ribosomal RNA genes, 22 transfer RNA genes, one light-strand origin of replication, and one control region, with lengths of 16,528 bp and 16,533 bp, respectively. The mitogenome architecture, nucleotide composition, and codon usage of protein-coding genes were almost identical between the two *Exostoma* species, although some estimated parameters varied. Phylogenetic analysis strongly supported the monophyly of *Exostoma* in the subfamily Glyptosternae, and *Exostoma tibetanum* had the closest relationship to *Exostoma tenuicaudatum*. The divergence time estimation demonstrated that these two species diverged approximately 1.51 Ma during the early Pleistocene, which was speculated to be triggered by the river system changes caused by the uplift of the southeastern Tibetan Plateau. Selection pressure analyses indicated that all protein-coding genes of *Exostoma* species underwent a strong purifying selection, while minority positive sites from NADH dehydrogenase complex genes were detected. These findings are expected to promote our understanding of the molecular phylogeny of the genus *Exostoma* and provide valuable mitogenomic resources for the subfamily Glyptosternae.

## 1. Introduction

The mitochondria are important semi-autonomous organelles in eukaryotic cells as their main venues of aerobic respiration, and generally the main energy production center of ATP for eukaryotes. The typical mitochondrial genome (mitogenome) is a closed-ring and double-stranded compact DNA molecule. For vertebrates, the length of the mitogenome ranges from approximately 15 to 20 kb, and comprises 13 protein-coding genes (*PCGs*), two ribosomal RNA genes (*rRNAs*), 22 transfer RNA genes (*tRNAs*), one light-strand origin of replication (*O_L_*), and one control region (*D–loop*) [1,2]. Owing to the extrachromosomal genome with unique characteristics—such as simple architecture, low-level recombination, maternal inheritance, a relatively high evolutionary rate, and conserved gene components—the mitogenome is an invaluable tool widely applied in the fields of modern biology, including taxonomy, population genetics, phylogenetics, biodiversity, and genetic diseases, among others [3,4].

The subfamily Glyptosternae is a specialized group of catfishes belonging to the order Siluriformes and family Sisoridae, and is found primarily in the Tibetan Plateau and adjacent drainage basins. The genus *Exostoma* Blyth 1860—a clade of glyptosternine catfishes—can be distinguished by their having a continuous post-labial groove, flattened ventral surface of the body with horizontally patulous paired fins specified for adhesion, and distally flattened oar-shaped homodont dentition in two separated patches on both the upper and lower jaws [5,6]. Fishes of this genus generally dwell beneath the rocks in torrential streams and rivers, with a distribution range across from the Yarlung Tsangpo–Brahmaputra River drainage eastwards to the Chao Phraya River drainage in northwestern Thailand [7]. So far, a total of 20 species of *Exostoma* have been described and validated [8]. In the lower reaches of the Yarlung Tsangpo, the river rounds the eastern part of the Himalayas and turns south, forming the largest canyon in the world—the Yarlung Tsangpo Grand Canyon, where two valid species of *Exostoma* have been recorded, namely, *Exostoma tibetanum* and *Exostoma tenuicaudatum* [9].

To date, the complete mitogenome sequences have been determined for dozens of glyptosternine catfishes. However, very few documents of the mitogenomics of *Exostoma* species have been published [10]. Therefore, the complete mitogenomes from species of *Exostoma* are required to improve the accuracy of mitogenome features of this genus, as well as to promote the analysis of their phylogenetic positioning and evolutionary history. Keeping this background in mind, herein, we first sequenced, annotated, and characterized the complete mitogenome sequences of two sympatric *Exostoma* species distributed in the lower Yarlung Tsangpo River drainage, and then analyzed the mitogenome features of both species with respect to genome architecture, nucleotide composition, and the codon usage of *PCGs*. Then, we performed the phylogenetic analysis to determine the relationship of the genus *Exostoma* within the subfamily Glyptosternae based on the concatenated *PCG* sequences, as well as to estimate the divergence time and the gene selection pressure of this genus.

## 2. Materials and Methods

### 2.1. Sampling and DNA Extraction

The samples of *Exostoma tibetanum* and *Exostoma tenuicaudatum* were collected from the Xigong River—a tributary of the lower Yarlung Tsangpo River in Motuo County, southeast Tibet, China (90.201° E, 29.247° N, 670 m elevation). After collection, the muscle tissues were taken and preserved in dehydrated alcohol and stored at −20 °C. Total genomic DNA was extracted from muscle tissue using the TSP201 Animal Tissue DNA Extraction Kit (Tsingke, Wuhan, China) according to the manufacturer’s instructions. The concentration and quality of DNA were assessed by agarose gel electrophoresis.

### 2.2. Next-Generation Sequencing and Libraries’ Construction

Extracted genomic DNA was sent to Novogene (Beijing, China) and prepared for sequencing on the Illumina HiSeq platform (Illumina Inc., San Diego, CA, USA). High-quality DNA samples were randomly broken into fragments for paired-end sequencing (2 Â 150 bp), and DNA libraries were constructed according to the procedure of Illumina DNA library construction. Approximately 4013 Mb of raw data for *Exostoma tibetanum* and 4126 Mb for *Exostoma tenuicaudatum* were generated. The raw sequencing reads were trimmed and filtered using SOAPnuke to remove adapter contamination and low-quality reads, with a cutoff of Phred quality scores of Q20. Then, about 3999 and 4104 Mb of clean data were obtained for *Exostoma tibetanum* and *Exostoma tenuicaudatum*, respectively.

### 2.3. Mitogenome Assembly, Annotation, and Analysis

Assemblies of the clean reads obtained de novo were conducted using SPAdes 3.9 [11] to obtain the complete circular mitogenomes. The annotations were conducted using the MitoZ toolkit [12] according to the vertebrate mitochondrial codon system, and then checked manually with reference to the published mitogenome of *Exostoma* gaoligongense [10]. Composition of nucleotides, relative synonymous codon usage (RSCU), and interspecific genetic divergence were calculated using Mega 7.0 [13]. The nucleotide composition skew values were determined using the formulae AT skew = (A − T)/(A + T) and GC skew = (G − C)/(G + C) [14]. The RSCU value for each codon indicates the ratio of the observed frequency divided by its expected frequency under equal usage among the amino acids. The physical mitochondrial maps of both species were drawn using the CGView online server [15].

### 2.4. Phylogenetic Analysis

The mitogenomes of 18 glyptosternine catfishes, covering 9 genera obtained from the NCBI GenBank—including the two new mitogenomes determined in the present study—were used to perform the phylogenetic analysis. *Bagarius yarrelli*, *Glyptothorax annandalei*, and *Pseudecheneis sulcatus* were selected as the outgroup. Their distribution information, GenBank accession numbers, and mitogenome size are given in Table 1. Prior to the phylogenetic analysis, substitution saturation tests for each aligned *PCG* and the three codon positions were conducted in DAMBE v6 by comparing the index of substitution saturation (*I*_ss_) against the critical *I*_ss_ (referred as *I*_ss.c_) at which the sequences began to fail to recover the true tree [16]. Then, the 13 *PCGs* were concatenated to a combination of sequences set without a termination codon to detect the phylogenetic relationships between the two *Exostoma* species and their congeners. Moreover, we also compared the topologies of each *PCG* and the concatenated *PCGs* using the Shimodaira–Hasegawa test from the “phangorn” package in R software to examine the statistical significance of alternative topologies [17,18].

Phylogenetic trees inferred from concatenated *PCGs* were determined using Bayesian inference (BI) and maximum likelihood (ML) methods. The best-fit nucleotide substitution model was identified utilizing the corrected Akaike information criterion with PartitionFinder v2.0 on the PhyloSuite platform [19]. The BI analysis was implemented in MrBayes v3.2 [20], with the parameter block generated in PartitionFinder. In the BI analysis, the starting trees were random. Two independent analyses with four simultaneous Markov chain Monte Carlo (MCMC) runs of 10 million generations were made, sampling every 100 generations, for a total of 100,001 trees. The first 50,000 trees were discarded as burn-in. The consensus tree and posterior probabilities of the phylogeny were obtained from the subsequent 50,001 trees. The ML analysis was carried out in RAxML v8 [21] using the GTRGAMMAI model, based on a generated scheme block, with 1000 nonparametric bootstrap replicates to evaluate the node support values.

### 2.5. Divergence Time Estimation

The divergence time of *Exostoma* species was estimated based on a dataset of 13 *PCG* sequences with a relaxed molecular clock method in the program BEAST v.1.10 [22]. The fossil record of *Bagarius yarrelli* (5.3~1.8 Ma) was set as the calibration point [23]. A Yule process was implemented for the prior tree model, and the substitution model was selected as GTR + G + I based on codon partition. The log-normal relaxed model with rate of 0.01 was applied in the molecular clock block. All of the other model parameters were set to their prior defaults. Two independent runs of 500 million generations of Markov chains were set up to guarantee the uniformity of the results, sampling once every 5000 generations and abandoning the initial 10% of samples as burn-in. Tracer v1.8 was used to check the convergence of the chains to a stationary distribution and large effective sample size (ESS > 200) [24]. The consensus divergence tree with a node height distribution was generated using TreeAnnotator v1.8 in the BEAST package [25], and was visualized using FigTree v.1.4 [26].

### 2.6. Selection Pressure Analysis

The natural selection acting on *PCGs* is often characterized by the nonsynonymous-to-synonymous substitution ratio (dN/dS, referred to as *ω*), which is an effective indicator applied for detecting the direction and magnitude of selective pressure on amino acid substitution. The *ω* values greater than 1, equal to 1, and less than 1 indicate purifying selection, neutral evolution, and positive selection, respectively [27,28]. To calculate lineage-specific substitution rates for three *Exostoma* species, the codeml program with the branch model (free-ratio model parameters) was performed for each *PCG* and the concatenated *PCGs* in EasyCodeML [29], which could separately estimate the dN, dS, and *ω* values for each clade on the tree. Furthermore, in order to detect the positive selection on certain codons instead of entire genes, we applied the branch-site model with a likelihood ratio test under the null hypothesis of the substitution rate *ω* fixing to 1. To be specific, we respectively set *Exostoma tibetanum*, *Exostoma tenuicaudatum*, and *Exostoma gaoligongense* to the foreground, which computed the *ω* value for each codon in 13 *PCGs* of 3 species.

## 3. Results and Discussion

### 3.1. Mitogenome Architecture and Composition

**Figure 1:**
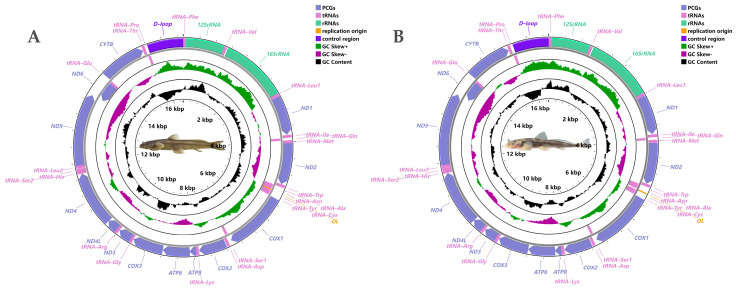
Physical mitogenome maps of *Exostoma tibetanum* (**A**) and *Exostoma tenuicaudatum* (**B**), drawn to scale, as indicated by the innermost circle. Genes encoded on the heavy or light strand are shown outside or inside of the outermost circle, respectively (arrows indicate the direction of gene transcription). The intermediate purple–green ring indicates the GC skewness (purple and green peaks indicate the positive and negative values, respectively). The GC content is plotted using a black ring, showing the deviation from the average GC content; PCGs—protein-coding genes, tRNAs—transfer RNA genes, rRNAs—ribosome RNA genes.

The complete mitochondrial genome of *Exostoma tibetanum* (GenBank accession no. ON641840) and *Exostoma tenuicaudatum* (GenBank accession no. ON641841) was 16,528 and 16,533 bp in length, respectively. The mitogenome content, gene arrangement, and gene-coding strands of both species were in accordance with the vertebrate consensus [30,31,32], and contained 13 *PCGs*, two *rRNAs*, 22 *tRNAs*, one *D–loop*, and one *O_L_*. The total length of the *PCGs* was 11,411 bp, accounting for 69.0% of the entire mitogenome for both species. Of all of the components, one *PCG* (*ND6*), eight *tRNAs* (i.e., *Gln*, *Ala*, *Asn*, *Cys*, *Tyr*, *Ser ^1^*, *Glu*, *Pro*), and *O_L_* were located on the light (−) strand, while the remaining genes were encoded on the heavy (+) strand (Figure 1). There were 10 gene overlaps (−10~−1 bp in length) and 12 intergenic spacers (1~21 bp in length) observed in the mitogenomes for both *Exostoma tibetanum* and *Exostoma tenuicaudatum* (see Table S1 for details).

Nucleotide composition analysis showed that both mitogenomes displayed a slight bias towards base A over T, with overall A + T content of 54.8% in *Exostoma tibetanum* and 55.2% in *Exostoma tenuicaudatum*. The content of A + T in different regions for *Exostoma tibetanum* was 54.4% (*PCGs*), 53.8% (*rRNAs*), 55.6% (*tRNAs*), and 62.4% (*D–loop*); while it was 54.8% (*PCGs*), 54.4% (*rRNAs*), 56.4% (*tRNAs*), and 59.6% (*D–loop*) for *Exostoma tenuicaudatum*. For *PCGs*, to be specific, the second and third codons were also A/T-biased in nucleotide composition, except for the first codon (48.1% for both species). This base pattern is consistent with most previously reported mitogenomes of Siluriformes catfishes [33,34,35]. In addition, the skew metrics of both mitogenomes indicated that the AT skewness value of each region was positive from 0.058 to 0.432 for *Exostoma tibetanum* and 0.013 to 0.523 for *Exostoma tenuicaudatum*, except for the second codon position of the *PCGs*, which implied that A had more content than T (−0.371 for *Exostoma tibetanum* and −0.367 for *Exostoma tenuicaudatum*). In contrast, except for the CG skewness for the *tRNA* regions close to zero (0.0009 for *Exostoma tibetanum* and −0.002 for *Exostoma tenuicaudatum*), all of the other CG skewness values were negative—from −0.631 to −0.042 for *Exostoma tibetanum*, and from −0.635 to −0.050 for *Exostoma* *tenuicaudatum*—indicating a higher content of C than G (Table 2).

### 3.2. Protein-Coding Genes and Codon Usage

In the mitogenomes of the two *Exostoma* species, 13 *PCGs* comprise a total of 3790 codons for both *Exostoma tibetanum* and *Exostoma tenuicaudatum*. Most of the *PCGs* use ATG as the start codon, in addition to *COX1* using GTG as the start codon. The majority of the PCGs use TAR (R represents A or G) as the stop codon, whereas *ND6* of *Exostoma tibetanum* uses AGA as the stop codon, and four genes (*COX2*, *COX3*, *NAD4*, and *CYTB*) exhibit incomplete stop codons (T or TA) (Table S1). Despite the close evolutionary relationship of the two *Exostoma* species, their mitochondrial genomes exhibited slightly varied stop codons. An incomplete stop codon in these genes is also a common feature in other reported species of vertebrates [32,36].

**Figure 2:**
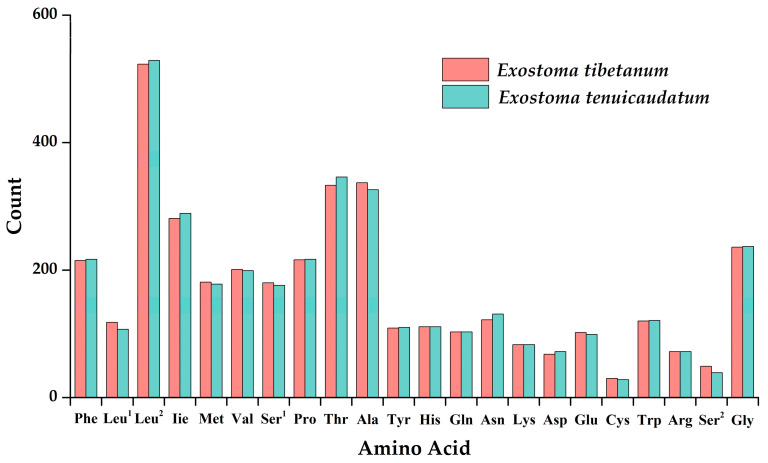
Amino acid frequency in the protein-coding genes of *Exostoma tibetanum* and *Exostoma tenuicaudatum*.

**Figure 3:**
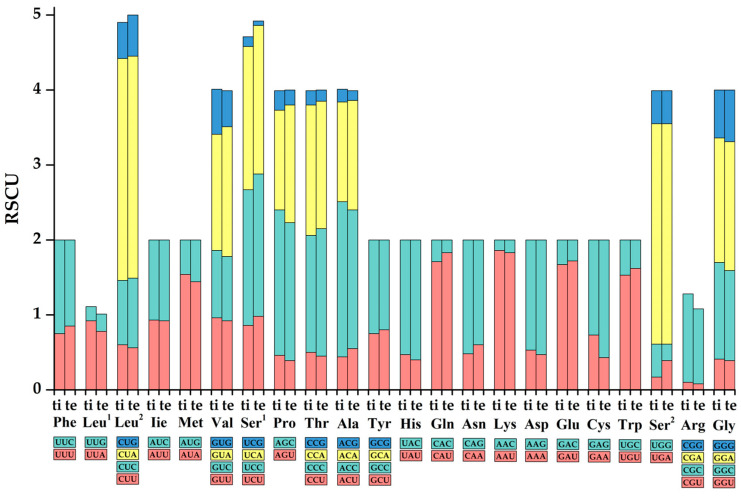
The base composition and the relative synonymous codon usage values of *Exostoma tibetanum* (ti) and *Exostoma tenuicaudatum* (te). Blue, yellow, green, and pink represent the first, second, third, and fourth type of each amino acid, respectively.

Both mitogenomes showed coincident distribution patterns for the concatenated *PCGs* codons, in spite of a few divergences (Figure 2, Table S2). The most frequently used codons for *Exostoma tibetanum* were CUN (*Leu^2^*) with 523 times, GCN (*Ala*) with 337 times, and UAY (*Tyr*) with 333 times, whereas the most frequently used codons in *Exostoma tenuicaudatum* were CUN (*Leu^2^*) with 529 times, GCN (*Ala*) with 325 times, and AUY (*Ile*) with 291 times. UGY (*Cys*) was the least used *PCG* codon, with 30 times in *Exostoma tibetanum* and 31 times in *Exostoma tenuicaudatum*. Moreover, the RSCU values of the 13 *PCGs* for both mitogenomes were calculated, and are visualized in Figure 3. The codon usage was biased to A/U over G/C at the third codon positions, which is similar to most mitogenomes of teleosts [31,37]. This phenomenon might be associated with the nucleotide composition, tRNA abundance, gene expression level, protein function, etc. [38,39].

### 3.3. Phylogenetic Analysis and Divergence Time Estimation

**Figure 4:**
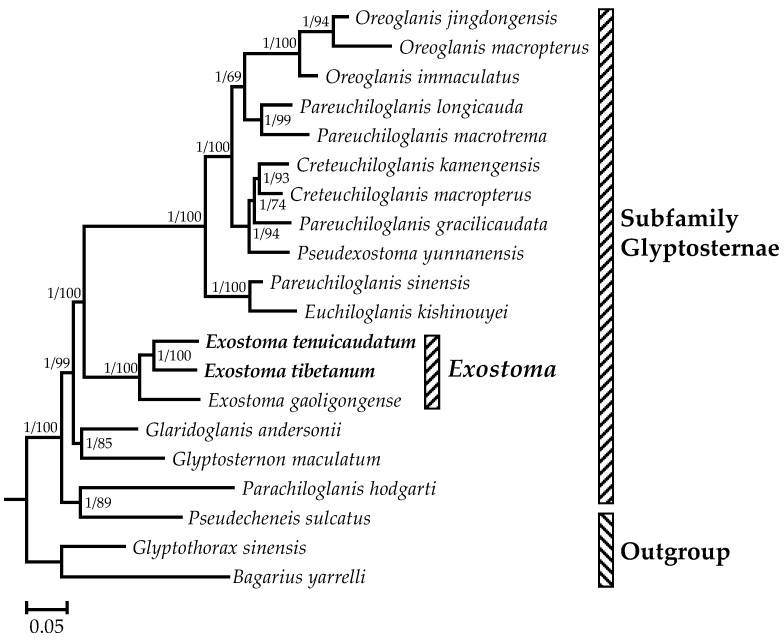
The phylogenetic trees constructed based on 13 protein-coding genes using Bayesian inference and maximum likelihood methods. Bayesian percent posterior probabilities and maximum likelihood bootstrap values are indicated at each node in the tree, separated by ‘/’.

For each *PCG* and the concatenated *PCGs*, the test of substitution saturation indicated that the values of *I*_ss_ were all significantly smaller than *I*_ss.c_ (*p* < 0.01), suggesting no saturation in the *PCGs* dataset, rendering it suitable for phylogenetic analysis. First, phylogenetic trees were generated based on the 13 single *PCGs* and the concatenated dataset. The Shimodaira–Hasegawa test suggested that the 13 topologies inferred from single *PCGs* showed a significant reduction (*p* < 0.01) in log-likelihood (ln *L*), and were statistically different from the tree topology inferred from concatenated *PCGs* (Table S3). Meanwhile, the phylogenetic trees generated from BI and ML analyses based on the 13 concatenated *PCGs* showed highly congruent topology. Therefore, only the BI tree with posterior probabilities and bootstrap values from ML analysis was given (Figure 4). As the results showed, on the overall scale, at the base of the phylogenetic tree, *Bagarius yarrelli* clustered with *Glyptothorax sinensis*, and then clustered with the clade containing *Pseudecheneis sulcatus* and *Parachiloglanis hodgarti*, indicating that the monophyly of glyptosternine fishes was undetermined under the definition of He et al. [40] inferred from morphological traits. Similar conclusions have also been drawn by Ma et al. [34]. In addition, the currently defined genus *Pareuchiloglanis* is not a monophyletic group, and could be divided into at least three different clades under current species coverage. The topology of the phylogenetic tree demonstrated that species of the genus *Exostoma* form a highly supported (1/100) monophyletic group in the subfamily Glyptosternae. Specifically, *E tenuicaudatum* is a sister clade to its sympatric congener *Exostoma tibetanum*, with a Kimura two-parameter distance of 9.2%. Then, both species were clustered with *Exostoma gaoligongense*, and next grouped with species of other glyptosternine genera.

**Figure 5:**
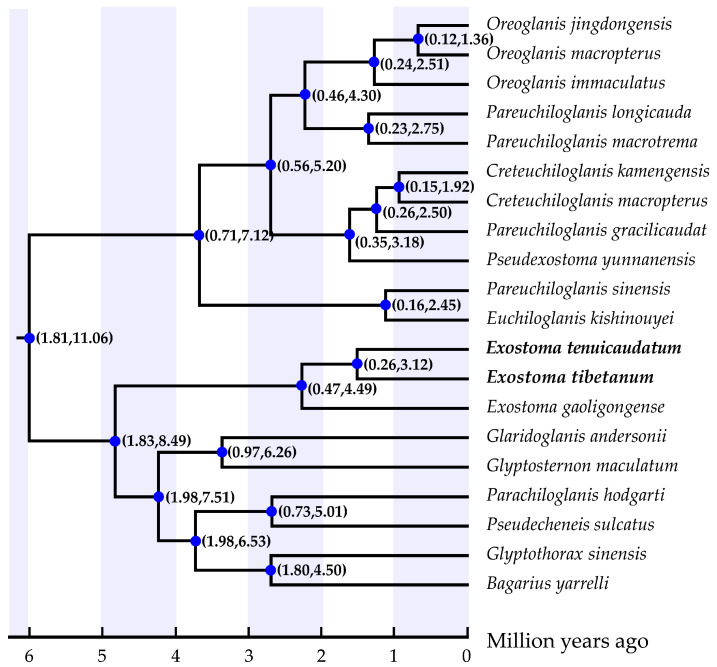
Divergence time tree of the glyptosternine catfishes derived from the partitioned Bayesian analysis using the relaxed molecular clock method in BEAST. Numbers on nodes (in brackets) indicate the 95% highest posterior density interval of divergence time.

The divergence time with 95% highest posterior density interval (95%HPD) estimated for the most recent common ancestor of *Exostoma tibetanum and Exostoma tenuicaudatum* arose 1.51 Ma (95%HPD: 0.25–3.12 Ma) during the early Pleistocene. The divergence time of *Exostoma gaoligongense* and the two aforementioned species was computed at 2.27 Ma, with a 95%HPD of 0.47–4.49 Ma (Figure 5). These results are later than the estimation of Ma et al. [34], who dated the common ancestor of *Exostoma* species to 5.52 Ma during the late Miocene (5.33–23.03 Ma). This discrepancy may account for the inclusion of more *Exostoma* species in the present study so as to determine the accurate topology of the evolutionary tree. Peng et al. [41] concluded that the origin and diversification of glyptosternine fishes were shaped by the three rapid uplift and two long-term peneplanation events in the Tibetan Plateau, as well as the river capture and reversal events resulting from the orogenic movement in eastern Tibet during the Miocene. Clark et al. [42] indicated that the ancient Yarlung Tsangpo River was once connected with other river drainages (i.e., the Irrawaddy River and Lohit River) in the Holocene (~4 Mya). The formation of the current river system pattern and formation of the Yarlung Tsangpo Grand Canyon were determined by at least two river capture events, accompanied by the rapid uplift of the Tibetan Plateau [43,44]. It is in this period that many endemic glyptosternine fishes—including species of *Exostoma*—branched off from their ancestors, facilitating the unique glyptosternine fish diversity patterns in this region.

### 3.4. Selection Pressure Analysis

**Figure 6:**
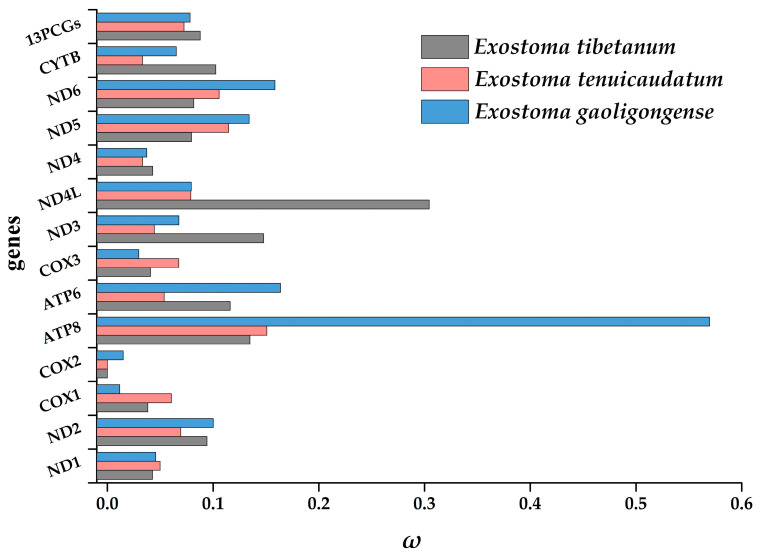
The ratio of non-synonymous substitutions to synonymous substitutions (*ω*) for each protein-coding gene and concatenated genes of *Exostoma tibetanum*, *Exostoma tenuicaudatum*, and *Exostoma gaoligongense*.

The separate *ω* values for the three *Exostoma* species yielded from the branch model in the codeml program showed that the *ω* values of the 13 *PCGs*, as well as the concatenated genes, were all less than 1, ranging from 0.0001 (*COX2* of *Exostoma tibetanum* and *Exostoma tenuicaudatum*) to 0.5699 (*ATP8* of *Exostoma gaoligongense*) (Figure 6, see Table S4 for details). This phenomenon of general purifying selection of *PCGs* was consistent with the signature of strong functional constraints of mitochondria as the main energy production center in eukaryotes, which could eliminate deleterious mutations to prevent the decrease in fitness, and was usually detected in other teleosts [45,46,47]. To be specific, the *ATP8* gene exhibited the highest *ω* value, similar to other eukaryotic organisms [48,49,50]. The *COX1* gene had the lowest value, showing that it was the most conserved gene. Lower *ω* values represent less variation in amino acids. Therefore, the genes with the lowest values (e.g., *COX1*, *COX2*, and *COX3*) are potential barcoding markers for the delimitation of *Exostoma* species. Ma [34] has indicated that the *ω* values of basal glyptosternine fishes are lower than those of specialized species, implying that this group of species has undergone rapid evolution of the subfamily Glyptosternae through the dispersal process. The estimation results of this study reveal that *Exostoma* is a relatively old fish group in the subfamily Glyptosternae, which is consistent with Ma’s conclusions.

Under the branch-site model applied for searching the positively selective sites from the purifying selective genes, two positive sites were detected, as follows: When we set *Exostoma tibetanum* as the foreground, the model detected moderately significant output of the positive site at *ND1*, with residue 103 as a positively selective site at a 90% confidence level. When *Exostoma gaoligongense* was selected as the foreground, the model determined significant evidence at *ND6*, with residue 46 deemed to be a positively selected site at a 95% confidence level (Table 3). Both *ND1* and *ND6* belong to the NADH dehydrogenase complex, which primarily contributes to the OXPHOS pathway in eukaryotic cells. This process produces approximately 40% of the proton pumps providing for ATP synthesis [51,52]. Previous studies revealed a similar phenomenon that positively selective sites were more likely to be found in the NADH complex genes, which could account for the less conserved protein function [53,54]. These positive selections may influence the modulation of mitochondrial complexes and electron transport efficiency and, hence, could facilitate their adaptive changes in response to cold and hypoxic water environments in the Tibetan Plateau, which has been confirmed in some Tibetan mammals and loaches [55,56,57]. Furthermore, more information on the structure, function, and evolution at the transcriptome and nuclear genome levels, as well as genome editing experiments for glyptosternine catfishes, are required to reveal the molecular mechanisms that underlie adaptations to high-altitude survival.

## 4. Conclusions

In this study, we sequenced and annotated the mitogenomes of two sympatric catfishes of *Exostoma* (*Exostoma tibetanum* and *Exostoma tenuicaudatum*) for the first time. The circular mitogenomes displayed lengths of 16,528 bp and 16,533 bp, respectively. Both mitogenomes encoded a typical set of 37 genes with conservative gene arrangement, which contained 13 *PCGs*, 2 *rRNAs*, and 22 *tRNAs*, together with one *O_L_* and one *D–loop*. Additionally, the nucleotide composition, skew values, codon usage, and RSCU of the 13 *PCGs*, were comparatively analyzed. Phylogenetic analysis inferred from the *PCG* sequences demonstrated that three included *Exostoma* species are clustered into monophyletic clades nested within the subfamily Glyptosternae, of which *Exostoma tibetanum* is a sister clade to *Exostoma tenuicaudatum*. Estimation of divergence time revealed that the speciation of both sympatric species might have been triggered by river system changes in the lower Yarlung Tsangpo River, accompanied by the uplift movement in the southeastern Tibetan Plateau during the early Pleistocene. The selective pressure analysis for *Exostoma* species demonstrated homogeneous purification selection for each gene, with several positively selective sites detected in NADH dehydrogenase complex genes, which may be associated with the adaptation to their cold and hypoxic habitats. The molecular data obtained could provide insights into the mitogenome architecture and molecular phylogeny of *Exostoma* fishes. In the future, more species of *Exostoma* should be included in the mitogenomics analysis to promote a better understanding of the molecular evolutionary processes of this genus.

## Figures and Tables

**Table 1:** Sample distribution information, GenBank accession numbers, and mitogenome size of the species included in the phylogenetic analysis.

Species	Drainage	GenBank No.	Size (bp)
*Bagarius yarrelli*	Irrawaddy River	NC021606	16,503
*Creteuchiloglanis kamengensis*	Yarlung Tsangpo River	MN396886	16,589
*Creteuchiloglanis macropterus*	Nujiang River	KP872683	16,589
*Euchiloglanis kishinouyei*	Jinsha River	NC021598	16,561
*Exostoma gaoligongense*	Nujiang River	NC056351	16,529
*Exostoma tibetanum*	Yarlung Tsangpo River	ON641840	16,528
*Exostoma tenuicaudatum*	Yarlung Tsangpo River	ON641841	16,533
*Glaridoglanis andersonii*	Lohit River	NC021600	16,532
*Glyptosternon maculatum*	Yarlung Tsangpo River	NC021597	16,539
*Glyptothorax sinensis*	Yangtze River	KJ739617	16,531
*Oreoglanis immaculatus*	Irrawaddy River	NC028511	16,576
*Oreoglanis jingdongensis*	Irrawaddy River	NC028512	16,569
*Oreoglanis macropterus*	Nujiang River	NC021607	16,568
*Parachiloglanis hodgarti*	Yarlung Tsangpo River	MW715684	16,511
*Pareuchiloglanis gracilicaudata*	Lancang River	NC021603	16,588
*Pareuchiloglanis longicauda*	Pearl River	NC028514	16,535
*Pareuchiloglanis macrotrema*	Red River	NC028515	16,570
*Pareuchiloglanis sinensis*	Jinsha River	KP872695	16,572
*Pseudecheneis sulcatus*	Yarlung Tsangpo River	MK843301	16,535
*Pseudexostoma yunnanensis*	Irrawaddy River	JQ026258	16,598

**Table 2:** A + T contents and AT/GC skewness of the mitochondrial genomes of *Exostoma tibetanum* and *Exostoma tenuicaudatum*.

	A + T (%)		G + C (%)		AT Skewness		GC Skewness	
	ti	te	ti	te	ti	te	ti	te
Whole mitogenome	54.8	55.2	45.2	44.8	0.135	0.138	–0.256	–0.315
Protein-coding genes	54.4	54.8	45.6	45.2	0.058	0.066	–0.325	–0.330
1st codon position	48.1	48.1	51.9	51.9	0.152	0.168	–0.042	–0.050
2nd codon position	58.5	58.7	41.5	41.3	–0.371	–0.365	–0.354	–0.363
3rd codon position	56.6	57.7	43.4	42.3	0.432	0.411	–0.631	–0.641
rRNA genes	53.8	54.4	46.2	45.6	0.279	0.283	–0.156	–0.155
tRNA genes	55.6	56.4	44.4	43.6	0.065	0.046	0.009	–0.002
Control region	62.4	59.6	37.6	40.4	0.071	0.013	–0.186	–0.094

ti represents *Exostoma tibetanum*; te represents *Exostoma tenuicaudatum*.

**Table 3:** Analysis based on the branch-site models for protein-coding genes of three *Exostoma* species.

Genes	Model	−LnL	Df.	Parameter Estimates	*p*-Value	Positive Sites
*ND1*	Fore_ti	−6879.48	1	f_0_ = 0.966, f_1_ = 0.030, f_2a_ = 0.004, f_2b_ = 0.000, ω_0_ = 0.040, ω_1_ = 1, ω_2_ = 13.576	0.062	103 N 0.982 *
	Null	−6881.22	1	ω_0_ = 0.040, ω_1_ = 1, ω_2_ = 1		
*ND6*	Fore_ga	−3759.45	1	f_0_ = 0.874, f_1_ = 0.110, f_2a_ = 0.015, f_2b_ = 0.002, ω_0_ = 0.044, ω_1_ = 1, ω_2_ = 2.740	0.030	46 H 0.961 *
	null	−3757.10	1	ω_0_ = 0.044, ω_1_ = 1, ω_2_ = 1		

Fore_ti indicates *Exostoma tibetanum* as the foreground in the branch-site model. Fore_ga indicates *Exostoma gaoligongense* as the foreground in the branch-site model.

## Data Availability

The mitochondrial genomes have been deposited at the NCBI GenBank under the accession numbers ON641840 and ON641841.

## Supplementary Materials

The following supporting information can be downloaded at: https://www.mdpi.com/article/10.3390/genes13091615/s1. Table S1: Gene or element features of the mitochondrial genomes of *Exostoma tibetanum* and *Exostoma tenuicaudatum*; Table S2: The codon counts and relative synonymous codon usage (RSCU) in the mitochondrial protein-coding genes of *Exostoma tibetanum* and *Exostoma tenuicaudatum*; Table S3: The differences in phylogenetic topologies inferred from concatenated protein-coding genes and single protein-coding genes for glyptosternine catfishes via the Shimodaira–Hasegawa test; Table S4: Estimation of dN, dS, and *ω* values for protein-coding genes of three *Exostoma* species.
