# Supplementary material for: Comparative Mitogenomics of Two Sympatric Catfishes of Exostoma (Siluriformes: Sisoridae) from the Lower Yarlung Tsangpo River and Its Application for Phylogenetic Consideration
Source: Genes (Basel). 2022 Sep 8;13(9):1615. doi: 10.3390/genes13091615 (PMC9498720; doi:10.3390/genes13091615)
Supplement: Supplementary file 1 [file genes-13-01615-s001.zip › genes-1853309-supplementary.pdf]

**Table S1.** Gene or element features of the mitochondrial genomes of *Exostoma tibetanum* and *Exostoma tenuicaudatum*.

| Gene | Strand | Location | | Size (bp) | | Start codon | | Stop codon | | Anticodon | | IGN | |
|---|---|---|---|---|---|---|---|---|---|---|---|---|---|
| | | ti | te | ti | te | ti | te | ti | te | ti | te | ti | te |
| *tRNA–Phe* | + | 1–69 | 1–69 | 69 | 69 | | | | | GAA | GAA | | |
| *12SrRNA* | + | 71–1024 | 71–1025 | 954 | 955 | | | | | | | 1 | 1 |
| *tRNA–Val* | + | 1025–1096 | 1026–1097 | 72 | 72 | | | | | TAC | TAC | 0 | 0 |
| *16SrRNA* | + | 1118–2768 | 1119–2770 | 1651 | 1652 | | | | | | | 21 | 21 |
| *tRNA–Leu*$^{1}$ | + | 2767–2841 | 2769–2843 | 75 | 75 | | | | | TAA | TAA | –2 | –2 |
| *ND1* | + | 2844–3815 | 2847–3818 | 972 | 972 | ATG | ATG | TAA | TAA | | | 2 | 3 |
| *tRNA–Ile* | + | 3821–3892 | 3824–3895 | 72 | 72 | | | | | GAT | GAT | 5 | 5 |
| *tRNA–Gln* | – | 3892–3962 | 3895–3965 | 71 | 71 | | | | | TTG | TTG | –1 | –1 |
| *tRNA–Met* | + | 3962–4032 | 3965–4035 | 71 | 71 | | | | | CAT | CAT | –1 | –1 |
| *ND2* | + | 4033–5079 | 4039–5085 | 1047 | 1047 | ATG | ATG | TAG | TAG | | | 0 | 3 |
| *tRNA–Trp* | + | 5080–5151 | 5086–5155 | 72 | 70 | | | | | TCA | TCA | 0 | 0 |
| *tRNA–Ala* | – | 5154–5222 | 5158–5226 | 69 | 69 | | | | | TGC | TGC | 2 | 2 |
| *tRNA–Asn* | – | 5224–5296 | 5228–5300 | 73 | 73 | | | | | GTT | GTT | 1 | 1 |
| $O_L$ | – | 5299–5328 | 5303–5332 | 30 | 30 | | | | | | | 2 | 2 |
| *tRNA–Cys* | – | 5329–5394 | 5333–5398 | 67 | 67 | | | | | GCA | GCA | 0 | 0 |
| *tRNA–Tyr* | – | 5395–5465 | 5399–5469 | 71 | 71 | | | | | GTA | GTA | 0 | 0 |
| *COX1* | + | 5467–7017 | 5471–7021 | 1551 | 1551 | GTG | GTG | TAA | TAA | | | 1 | 1 |
| *tRNA–Ser*$^{1}$ | – | 7018–7088 | 7022–7092 | 71 | 71 | | | | | TGA | TGA | 0 | 0 |
| *tRNA–Asp* | + | 7093–7162 | 7097–7166 | 70 | 70 | | | | | GTC | GTC | 4 | 4 |
| *COX2* | + | 7177–7867 | 7181–7871 | 691 | 691 | ATG | ATG | T | T | | | 14 | 14 |
| *tRNA–Lys* | + | 7868–7942 | 7872–7946 | 75 | 75 | | | | | TTT | TTT | 0 | 0 |
| *ATP8* | + | 7943–8110 | 7948–8115 | 168 | 168 | ATG | ATG | TAA | TAA | | | 0 | 1 |
| *ATP6* | + | 8101–8784 | 8106–8789 | 684 | 684 | ATG | ATG | TAA | TAA | | | –10 | –10 |
| *COX3* | + | 8784–9568 | 8789–9573 | 785 | 785 | ATG | ATG | TA | TA | | | –1 | –1 |
| *tRNA–Gly* | + | 9569–9635 | 9574–9642 | 67 | 69 | | | | | TCC | TCC | 0 | 0 |
| *ND3* | + | 9636–9986 | 9643–9993 | 351 | 351 | ATG | ATG | TAG | TAG | | | 0 | 0 |
| *tRNA–Arg* | + | 9987–10056 | 9994–10062 | 70 | 69 | | | | | TCG | TCG | 0 | 0 |
| *ND4L* | + | 10057–10353 | 10063–10359 | 297 | 297 | ATG | ATG | TAA | TAA | | | 0 | 0 |
| *ND4* | + | 10347–11727 | 10353–11733 | 1381 | 1381 | ATG | ATG | T | T | | | –7 | –7 |
| *tRNA–His* | + | 11728–11797 | 11734–11803 | 70 | 70 | | | | | GTG | GTG | 0 | 0 |
| *tRNA–Ser*$^{2}$ | + | 11798–11864 | 11804–11870 | 67 | 67 | | | | | GCT | GCT | 0 | 0 |
| *tRNA–Leu*$^{2}$ | + | 11871–11942 | 11875–11946 | 72 | 72 | | | | | TAG | TAG | 6 | 4 |
| *ND5* | + | 11943–13769 | 11947–13773 | 1827 | 1827 | ATG | ATG | TAA | TAA | | | 0 | 0 |
| *ND6* | – | 13765–14283 | 13770–14288 | 519 | 519 | ATG | ATG | AGA | TAA | | | –5 | –1 |
| *tRNA–Glu* | – | 14284–14352 | 14289–14357 | 69 | 69 | | | | | TTC | TTC | –3 | 0 |
| *CYTB* | + | 14355–15492 | 14360–15497 | 1138 | 1138 | ATG | ATG | T | T | | | 2 | 2 |
| *tRNA–Thr* | + | 15493–15564 | 15498–15569 | 72 | 72 | | | | | TGT | TGT | 0 | 0 |
| *tRNA–Pro* | – | 15563–15632 | 15568–15637 | 70 | 70 | | | | | TGG | TGG | –2 | –2 |
| *D–loop* | + | 15633–16528 | 15638–16533 | 896 | 896 | | | | | | | 0 | 0 |

ti representing *E. tibetanum*; te representing *E. tenuicaudatum*.

**Table S2.** The codon counts and relative synonymous codon usages (RSCU) in the mitochondrial protein-coding genes of *Exostoma tibetanum* and *Exostoma tenuicaudatum*.

| Amino acid | Codon | *E. tibetanum* | | *E. tenuicaudatum* | |
|---|---|---|---|---|---|
| | | Count | RSCU | Count | RSCU |
| *Phe* | UUU | 81 | 0.75 | 92 | 0.85 |
| | UUC | 134 | 1.25 | 125 | 1.15 |
| *Leu*[1] | UUA | 98 | 0.92 | 83 | 0.78 |
| | UUG | 20 | 0.19 | 24 | 0.23 |
| *Leu*[2] | CUU | 64 | 0.6 | 59 | 0.56 |
| | CUC | 92 | 0.86 | 98 | 0.92 |
| | CUA | 316 | 2.96 | 314 | 2.96 |
| | CUG | 51 | 0.48 | 58 | 0.55 |
| *Ile* | AUU | 131 | 0.93 | 133 | 0.92 |
| | AUC | 150 | 1.07 | 156 | 1.08 |
| *Met* | AUA | 139 | 1.54 | 128 | 1.44 |
| | AUG | 42 | 0.46 | 50 | 0.56 |
| *Val* | GUU | 48 | 0.96 | 46 | 0.92 |
| | GUC | 45 | 0.9 | 43 | 0.86 |
| | GUA | 78 | 1.55 | 86 | 1.73 |
| | GUG | 30 | 0.6 | 24 | 0.48 |
| *Ser*[1] | UCU | 33 | 0.86 | 35 | 0.98 |
| | UCC | 69 | 1.81 | 68 | 1.9 |
| | UCA | 73 | 1.91 | 71 | 1.98 |
| | UCG | 5 | 0.13 | 2 | 0.06 |
| *Pro* | CCU | 25 | 0.46 | 21 | 0.39 |
| | CCC | 105 | 1.94 | 100 | 1.84 |
| | CCA | 72 | 1.33 | 85 | 1.57 |
| | CCG | 14 | 0.26 | 11 | 0.2 |
| *Thr* | ACU | 42 | 0.5 | 39 | 0.45 |
| | ACC | 130 | 1.56 | 147 | 1.7 |
| | ACA | 145 | 1.74 | 147 | 1.7 |
| | ACG | 16 | 0.19 | 13 | 0.15 |
| *Ala* | GCU | 37 | 0.44 | 45 | 0.55 |
| | GCC | 174 | 2.07 | 151 | 1.85 |
| | GCA | 112 | 1.33 | 119 | 1.46 |
| | GCG | 14 | 0.17 | 11 | 0.13 |
| *Tyr* | UAU | 41 | 0.75 | 44 | 0.8 |
| | UAC | 68 | 1.25 | 66 | 1.2 |
| *His* | CAU | 26 | 0.47 | 22 | 0.4 |
| | CAC | 85 | 1.53 | 89 | 1.6 |
| *Gln* | CAA | 88 | 1.71 | 94 | 1.83 |
| | CAG | 15 | 0.29 | 9 | 0.17 |
| *Asn* | AAU | 29 | 0.48 | 39 | 0.6 |
| | AAC | 93 | 1.52 | 92 | 1.4 |
| *Lys* | AAA | 77 | 1.86 | 76 | 1.83 |
| | AAG | 6 | 0.14 | 7 | 0.17 |
| *Asp* | GAU | 18 | 0.53 | 17 | 0.47 |
| | GAC | 50 | 1.47 | 55 | 1.53 |
| *Glu* | GAA | 85 | 1.67 | 85 | 1.72 |
| | GAG | 17 | 0.33 | 14 | 0.28 |

| | | | | | |
|---|---|---|---|---|---|
| *Cys* | UGU | 11 | 0.73 | 6 | 0.43 |
| | UGC | 19 | 1.27 | 22 | 1.57 |
| *Trp* | UGA | 92 | 1.53 | 98 | 1.62 |
| | UGG | 28 | 0.47 | 23 | 0.38 |
| *Arg* | CGU | 3 | 0.17 | 7 | 0.39 |
| | CGC | 8 | 0.44 | 4 | 0.22 |
| | CGA | 53 | 2.94 | 53 | 2.94 |
| | CGG | 8 | 0.44 | 8 | 0.44 |
| *Ser*[2] | AGU | 4 | 0.1 | 3 | 0.08 |
| | AGC | 45 | 1.18 | 36 | 1 |
| *Gly* | GGU | 24 | 0.41 | 23 | 0.39 |
| | GGC | 76 | 1.29 | 71 | 1.2 |
| | GGA | 98 | 1.66 | 102 | 1.72 |
| | GGG | 38 | 0.64 | 41 | 0.69 |

**Table S3.** The differences of phylogenetic topologies inferred from concatenated protein-coding genes and single protein-coding gene for glyptosternine catfishes via Shimodaira–Hasegawa test.

| **Gene** | **Size (bp)** | **ln *L*** | **Diff ln *L*** | ***P*-value** |
|---|---|---|---|---|
| 13*PCGs* | 11379 | –99899.8 | 0 | 0.9638 |
| *ND1* | 972 | –119706 | 19806.228 | 0.0000 |
| *ND2* | 1044 | –103874.9 | 3975.136 | 0.0000 |
| *COX1* | 1545 | –117950.4 | 18050.58 | 0.0000 |
| *COX2* | 690 | –106545 | 6645.191 | 0.0000 |
| *ATP8* | 165 | –126735.7 | 26835.891 | 0.0000 |
| *ATP6* | 681 | –106094.6 | 6194.823 | 0.0000 |
| *COX3* | 783 | –109676.5 | 9776.72 | 0.0000 |
| *ND3* | 348 | –108157.9 | 8258.074 | 0.0000 |
| *ND4L* | 294 | –116608.9 | 16709.058 | 0.0000 |
| *ND4* | 1380 | –124910.1 | 25010.258 | 0.0000 |
| *ND5* | 1824 | –123050.7 | 23150.907 | 0.0000 |
| *ND6* | 516 | –123570.9 | 23671.09 | 0.0000 |
| *CYTB* | 1137 | –123448.8 | 23549.033 | 0.0000 |

**Table S4.** Estimation of dN, dS, and $\omega$ values for protein-coding genes of three *Exostoma* species.

| **genes** | ***Exostoma tibetanum*** | | | ***Exostoma tenuicaudatum*** | | | ***Exostoma gaoligongense*** | | |
|---|---|---|---|---|---|---|---|---|---|
| | **dN** | **dS** | $\omega$ | **dN** | **dS** | $\omega$ | **dN** | **dS** | $\omega$ |
| *ND1* | 0.0073 | 0.1699 | 0.0428 | 0.0148 | 0.2955 | 0.0500 | 0.0121 | 0.2630 | 0.0458 |
| *ND2* | 0.0255 | 0.2703 | 0.0944 | 0.0182 | 0.2618 | 0.0696 | 0.0252 | 0.2508 | 0.1004 |
| *COX1* | 0.0000 | 0.0000 | 0.0384 | 0.0000 | 0.0000 | 0.0607 | 0.0027 | 0.2317 | 0.0118 |
| *COX2* | 0.0000 | 0.1621 | 0.0001 | 0.0000 | 0.1660 | 0.0001 | 0.0041 | 0.2689 | 0.0151 |
| *ATP8* | 0.0238 | 0.1760 | 0.1350 | 0.0262 | 0.1736 | 0.1510 | 0.0711 | 0.1248 | 0.5699 |
| *ATP6* | 0.0172 | 0.1475 | 0.1164 | 0.0124 | 0.2307 | 0.0538 | 0.0378 | 0.2303 | 0.1640 |
| *COX3* | 0.0062 | 0.1504 | 0.0409 | 0.0095 | 0.1401 | 0.0677 | 0.0135 | 0.4521 | 0.0299 |
| *ND3* | 0.0352 | 0.2381 | 0.1480 | 0.0188 | 0.4230 | 0.0445 | 0.0353 | 0.5198 | 0.0678 |
| *ND4L* | 0.0169 | 0.0553 | 0.3046 | 0.0107 | 0.1357 | 0.0791 | 0.0206 | 0.2587 | 0.0795 |
| *ND4* | 0.0132 | 0.3067 | 0.0430 | 0.0102 | 0.3062 | 0.0333 | 0.0101 | 0.2691 | 0.0374 |
| *ND5* | 0.0195 | 0.2450 | 0.0797 | 0.0239 | 0.2078 | 0.1149 | 0.0286 | 0.2128 | 0.1343 |
| *ND6* | 0.0165 | 0.2015 | 0.0819 | 0.0227 | 0.2141 | 0.1059 | 0.0371 | 0.2341 | 0.1587 |
| *CYTB* | 0.0156 | 0.1519 | 0.1027 | 0.0119 | 0.3581 | 0.0333 | 0.0220 | 0.3373 | 0.0653 |
| 13 *PCGs* | 0.0132 | 0.1505 | 0.0880 | 0.0128 | 0.1761 | 0.0726 | 0.0189 | 0.2406 | 0.0784 |
